# Radiation Exposure, Knowledge, and Compliance in Upper Tract Urological Surgery: A Systematic Review

**DOI:** 10.7759/cureus.88278

**Published:** 2025-07-19

**Authors:** Shika Veera, William Olive, Vaishnavi J Patel, Kimberly C Toumazos, Virgil DeMario, Devki Patel, Young Son, Thomas J Mueller

**Affiliations:** 1 Department of Clinical Science, Rowan-Virtua School of Osteopathic Medicine, Stratford, USA; 2 Department of Clinical Science, Lincoln Memorial University DeBusk College of Osteopathic Medicine, Harrogate, USA; 3 Department of Family Medicine, The University of Texas at Austin Dell Medical School, Austin, USA; 4 Department of Urology, University of Kentucky College of Medicine, Lexington, USA; 5 Department of Clinical and Applied Science Education, University of the Incarnate Word School of Osteopathic Medicine, San Antonio, USA; 6 Office of Research and Innovation, Texas Tech University Health Sciences Center School of Medicine, Lubbock, USA; 7 Department of Urology, Jefferson Stratford Hospital, Stratford, USA; 8 Department of Urology, New Jersey Urology, LLC, Voorhees, USA

**Keywords:** education and training of medical students and doctors (specialist and phd), ionizing radiation, ionizing radiation exposure, personal protective equipment (ppe), ppe, quality improvement research, radiation exposure, systematic review, urological surgery, urology and uro oncology

## Abstract

The use of ionizing radiation in urology has grown significantly, increasing occupational risks to urologists. Despite established safety guidelines and protective equipment, adherence remains inconsistent due to limited education and systemic barriers. This review evaluates current practices in upper tract urological surgeries, identifies factors contributing to poor compliance, and explores strategies to enhance radiation safety in urology. Following the Preferred Reporting Items for Systematic Reviews and Meta-Analyses (PRISMA) guideline, 209 articles were screened, with 22 final studies meeting inclusion criteria. Two independent reviewers screened manuscripts to determine study eligibility, and a Cochrane Risk of Bias was performed. The findings revealed that trunk personal protective equipment (PPE) had the highest compliance, while eye and hand protection were the least used. An educational intervention led to a 99.9% increase in PPE compliance. Knowledge gaps were prevalent, with 63-64% of respondents reporting no formal training. Higher knowledge correlated with improved safety practices. PPE use significantly reduced radiation exposure: eyes (90%), hands (50%), thyroid (95%), and trunk (95%). Protocol modifications such as pulsed fluoroscopy and additional shielding were consistently associated with reduced exposure. These findings underscore the critical role of PPE and education in curtailing radiation exposure among urologists and surgical staff in upper tract urological surgery. Emphasizing proper training is paramount to instilling adherence to PPE protocols and implementing procedural techniques aimed at minimizing radiation exposure. The objective of this systematic review was to comprehensively assess existing literature, focusing on the efficacy of PPE, radiation safety protocols, educational initiatives, and procedural adaptations aimed at reducing radiation exposure among urological practitioners.

## Introduction and background

Over the past 40 years, the use of ionizing radiation, such as fluoroscopy and computed tomography, has increased significantly in the setting of upper tract endourological surgery. This is primarily driven by the increase in the global burden of kidney stone disease and the subsequent rise in the number of endourological interventions being performed, many of which utilize fluoroscopy. Upper tract urological surgeries include procedures such as percutaneous nephrolithotomy (PCNL) and ureteroscopy, which frequently involve the use of fluoroscopic imaging for stone visualization and treatment. While previous reviews have broadly discussed radiation exposure in endourology, none have specifically addressed upper tract urological surgery, which is uniquely characterized by longer fluoroscopy times and more complex imaging requirements. While these tools are essential for diagnosis and treatment, they pose occupational hazards to urologists, particularly in the absence of standardized personal protective equipment (PPE) [1]. Repeated radiation exposure can lead to serious health risks, including skin injuries, cataracts, and a heightened risk of malignancy [2]. In response, the International Commission on Radiological Protection (ICRP) introduced the concept of "As Low As Reasonably Achievable" (ALARA), which emphasizes justification, optimization, and dose limitation to minimize harm [3]. The ALARA principle is rooted in justification of radiation use, optimization of exposure through techniques such as pulsed fluoroscopy, and dose limitation via shielding and protective measures.

Despite established guidelines, radiation exposure among urologists remains highly variable. Although various interventions have been proposed to reduce exposure, including PPE and procedural modifications, their effectiveness is limited if not routinely and properly utilized [4]. Prior studies have reported inconsistent use of PPE, limited access to formal radiation safety education, and suboptimal adherence to safety protocols. Consistent with the ALARA principles, numerous studies have quantified radiation exposure across various urologic procedures, further highlighting the need for comprehensive evaluation and standardization [4]. One such systematic review from 2024 provided valuable insights into educational and training practices; however, it encompassed the broad field of urology, rather than focusing on upper tract urological surgery, and did not comprehensively discuss institutional policies, technological advances, or procedural factors influencing exposure [4]. Our review aims to address this paucity of data by providing a broader, multidimensional assessment of radiation safety in upper tract urological surgery, as a gap exists between recommendations and real-world practice, suggesting the presence of systemic barriers to implementation.

This review synthesizes current evidence on radiation safety in upper tract surgery, with an emphasis on identifying contributing factors to poor compliance and potential strategies to address them. By identifying knowledge gaps, analyzing behavioral trends, and addressing institutional shortcomings, this work aims to inform future training initiatives and foster a culture of safety within the field.

## Review

Literature search strategy

The review protocol was registered in PROSPERO: CRD42023404877 (International Prospective Register of Systematic Reviews). A systematic review was conducted in accordance with the Preferred Reporting Items for Systematic Reviews and Meta-Analyses (PRISMA) guidelines. PubMed, Embase, Scopus, and Web of Science were searched for studies published from 2013 to 2023 related to radiation safety in urology, including knowledge, education, PPE, and procedural modifications using a combination of keywords and MeSH terms: ("Radiation exposure"[MeSH Terms] OR ("radiation"[Title/Abstract] AND "exposure"[Title/Abstract])) AND ("urology"[MeSH Terms] OR "urology"[Title/Abstract]) AND ("procedures"[Title/Abstract] OR "endourology"[MeSH Terms] OR "scatter radiation"[Title/Abstract]) AND ("health outcomes"[Title/Abstract] OR "residents"[Title/Abstract] OR "residency"[Title/Abstract] OR "urologist"[Title/Abstract]).

**Figure 1:**
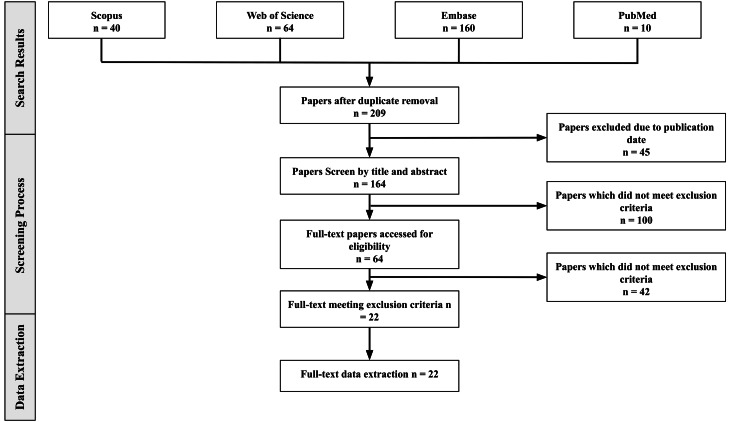
PRISMA Flowchart of the Included Literature From the Systematic Search PRISMA: Preferred Reporting Items for Systematic reviews and Meta-Analyses

After the removal of duplicates, 209 unique articles were screened. Two independent reviewers (SV and WO) screened titles and abstracts for relevance. Studies were included if they met the following criteria: (1) population of practicing or training urologists with potential radiation exposure; (2) focus on radiation knowledge, safety practices, PPE use, or radiation-reducing interventions in upper tract urological surgery; (3) published in English with full text available; and (4) peer-reviewed studies. Studies were excluded if they were case reports, case series, non-peer-reviewed articles, abstracts, graduate theses, conference presentations, more than 10 years old, or if they lacked complete data (Figure 1). Full-text screening and final inclusion were performed by two reviewers, with a third clinical adjudicator resolving any disagreements that arose. Data were synthesized thematically without the use of statistical software, and any discrepancies during study selection or data extraction were resolved through consensus among co-authors.

Data extraction and quality assessment

The study data were extracted using a standardized data collection form. This form was developed by the research team and piloted on a subset of included studies to ensure clarity and consistency. Variables extracted included study characteristics (e.g., author, year, study design, sample size), participant characteristics (e.g., profession, years of experience), radiation exposure details (e.g., type of procedure, exposure levels), PPE compliance, radiation safety knowledge, and safety measures to reduce radiation exposure. "Radiation knowledge" was quantified based on survey responses within the included studies, assessing understanding of radiation risks, safety principles (e.g., ALARA), and PPE use. Data on the percentage of correct answers, proportion of respondents aware of specific facts, or scores on knowledge scales were extracted, as reported in the individual studies. A qualitative and quantitative summary was performed for radiation dosage, reduction of radiation, and compliance.

Risk of bias

**Figure 2:**
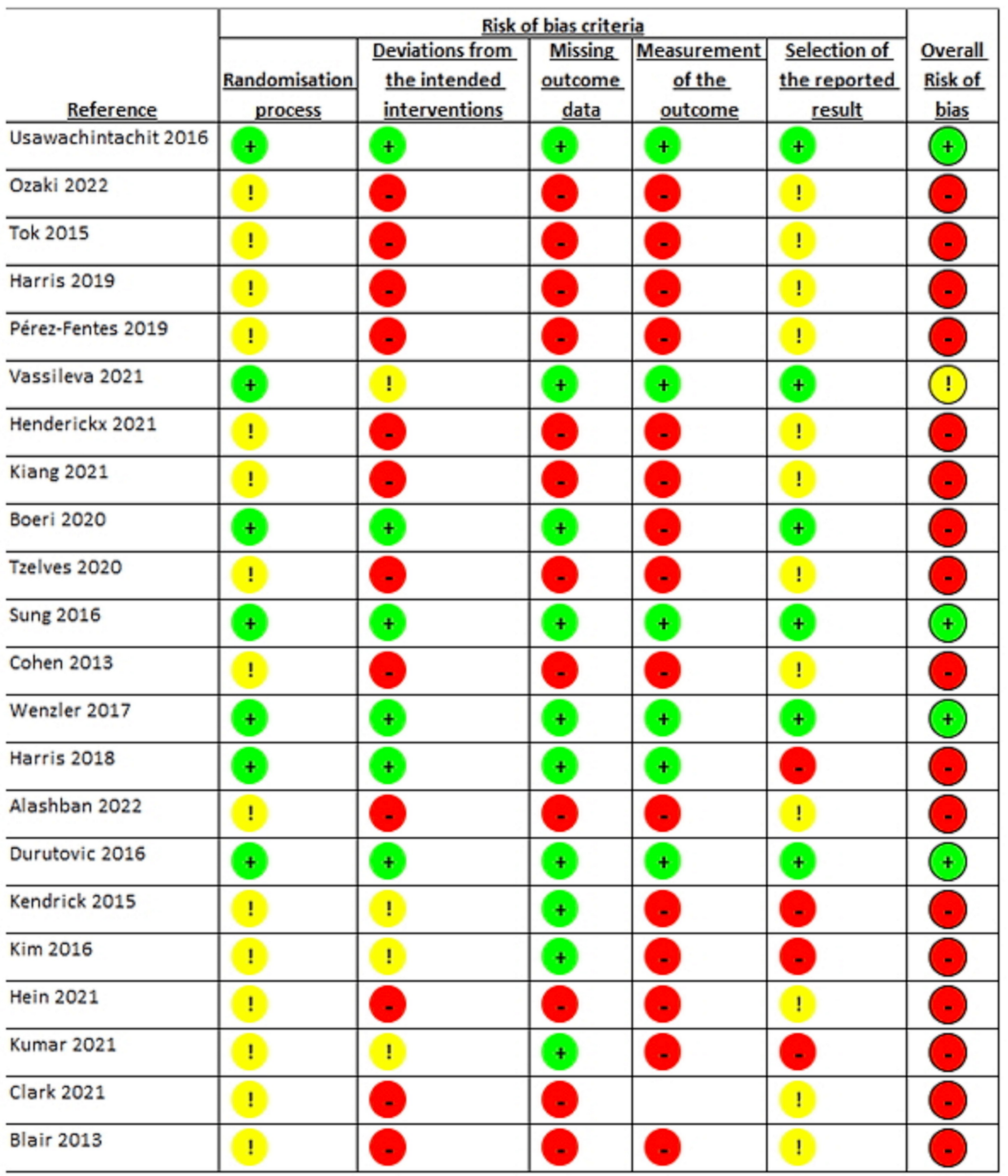
Cochrane Collaboration's Risk of Bias Criteria for the Included Literature in the Study Source: [2,5-25]

Risk-of-bias (RoB) assessment was performed independently by two authors (SV and WO) using the Cochrane Collaboration's RoB tool or the validated Joanna Briggs Institute Critical Appraisal Checklist for non-randomized studies. The majority of studies had an overall high RoB due to the deviation from intended interventions, missing outcome data, and measurement of the outcome. There were also a majority of studies with some concerns due to the randomization process and selection of the reported results. Most studies were conducted without blinding patients or personnel, which was due to the nature of the study designs (Figure 2).

Compliance

Eight studies assessed the compliance of staff, attending surgeons, and resident surgeons regarding the use of PPE for radiation exposure (Table 1). Across all studies, PPE covering the trunk was most commonly used, followed by thyroid PPE, with eye and hand PPE having the lowest compliance rates. In one study, participants were surveyed before and after an educational program about radiation safety [23]. There was a 99.9% increase in compliance with PPE following completion of the education program. Vassileva et al. conducted a prospective, multicenter study across six European countries to evaluate PPE usage during various endourological procedures, which complements the survey-based findings by providing observational data on real-world compliance patterns in a broader, international setting [9].

**Table 1:** Compliance Study PPE: personal protective equipment; US: United States; PCNL: percutaneous nephrolithotomy

Study	Type of Study	Sample Characteristics	Study Description	Compliance
Cohen et al. [15]	Retrospective case series	137 procedures, one surgeon	Analyzed data of all fluoroscopic stone removal procedures from a single experienced surgeon in nine months. The surgeon wore a lead apron, thyroid shield, and lead-lined glasses	Compliance for PPE of the eyes (9.7%), hands (17.2%), thyroid (68%), trunk and pelvis (97%)
Harris et al. [2]	Survey	All US Urology residents, 136 respondents	US urology residents were surveyed to assess their knowledge and training regarding radiation safety	Compliance for PPE for eyes (9%), hands (0%), thyroid (99%), trunk (97%)
Kumar et al. [23]	Prospective clinical trial	40 operating room staff were surveyed	Participants were given a survey before and after a structured educational program	99.9% increase in compliance to PPE and radiation exposure guidelines following the education program
Pérez-Fentes et al. [8]	Survey	238 respondents from the Spanish Association	Urologists in Spain were surveyed for radiation protection PPE compliance and awareness	Compliance for dosimeter badges of the eyes (2%), wrist (27%), and trunk (57%)
Tok et al. [7]	Survey	127 survey responses from urological surgical staff in Turkey	Surgical staff across all career levels and experience durations were surveyed to understand their understanding of radiation. Following education training, the staff were reassessed for knowledge and compliance	Post-education, there was an 18.6% improvement in compliance to radiation safety guidelines. This includes use of PPE for eyes (12%), hands (12%), thyroid (72.4%), trunk (92%), and dosimeters (46.5%)
Tzelves et al. [13]	Survey	211 respondents	Assessed compliance and understanding of radiation safety measures among endourologists and residents	Compliance for PPE of the eyes (14.7%), hands (8.1%), thyroid (84.4%), trunk (89.6%)
Vassileva et al. [9]	Prospective cohort study	315 procedures from six studies (international), 24 surgeons in six centers for three months	Centers in Turkey, Bulgaria, Greece, Italy, and Macedonia. All procedures analyzed were conducted between May and July 2018 and included five endourology procedures: PCNL, mini-PCNL, retrograde intrarenal surgery, semirigid ureteroscopy, and flexible ureteroscopy	All staff surveyed wore lead aprons and thyroid collars

Knowledge

Seven studies investigated the knowledge of radiation safety of operating room staff and urologists (Table 2). Overall, these studies showed that not everyone exposed to radiation was properly trained on the use of PPE and monitoring devices, such as dosimeters. A positive correlation was found between an individual’s knowledge of radiation and their increased compliance with safety measures. One study showed that 89% of urology resident respondents find radiation safety to be important, while only 46% reported that radiation safety was part of their training [11,15]. This study also showed that 64% of respondents desire more formal education about radiation safety. Similarly, another study showed that 63% of their respondents had no formal training in radiation safety [26]. At a baseline, 100% of respondents in one study answered correctly about the relationship between radiation exposure and PPE [15].

**Table 2:** Knowledge Variable US: United States; NVU and BVU: Dutch and Belgian associations of urology; ALARA: As Low As Reasonably Achievable

Study	Type of Study	Sample Characteristics	Study Description	Knowledge
Harris et al. [2]	Survey	US Urology residents and had 136 respondents	US urology residents were surveyed to assess their knowledge and training regarding radiation safety	The majority of respondents believed radiation safety is important (89%) and desire more formal education (64%)
Hein et al. [22]	Prospective multicenter study	303 patients	All retrograde intrarenal surgeries for removal of stones were compared retrospectively to prospectively after the staff received a presentation on radiation exposure	Following education, there was a 40.8% reduction in fluoroscopy time
Henderickx et al. [10]	Survey	170 respondents	A survey was sent to members of the NVU and BVU (Netherlands and Belgian national urology organizations) to assess radiation exposure awareness	Respondents unfamiliar with the ALARA principle (10%), did not take any additional radiation protection course (6%)
Kumar et al. [23]	Prospective clinical trial	40 operating room staff were surveyed	Participants were given a survey before and after a structured educational program	Education improved background knowledge of radiation risk by 62.5% and ALARA by 47.5%
Pérez-Fentes et al. [8]	Survey	Urologists in the Spanish Association of Urology and had 238 respondents	Urologists in Spain were surveyed for radiation protection PPE compliance and awareness	Respondents received no radiation protection training (63%), first-level accreditation (25%), second-level accreditation (12%)
Tok et al. [7]	Survey	127 survey responses from urological surgical staff in Turkey	Surgical staff were surveyed to assess their understanding of radiation. Following education training, the staff were reassessed for knowledge and compliance	Post-education, there was a 44.9% improvement in awareness of radiation exposure risks
Tzelves et al. [13]	Survey	211 respondents	A survey that assessed compliance and understanding of radiation safety measures among endourologists and residents	Aware of the ALARA principle (42%), believed radiation exposure increases malignancy risk (88%), mentioned having regular lessons (25%)

Risk to the body

**Figure 3:**
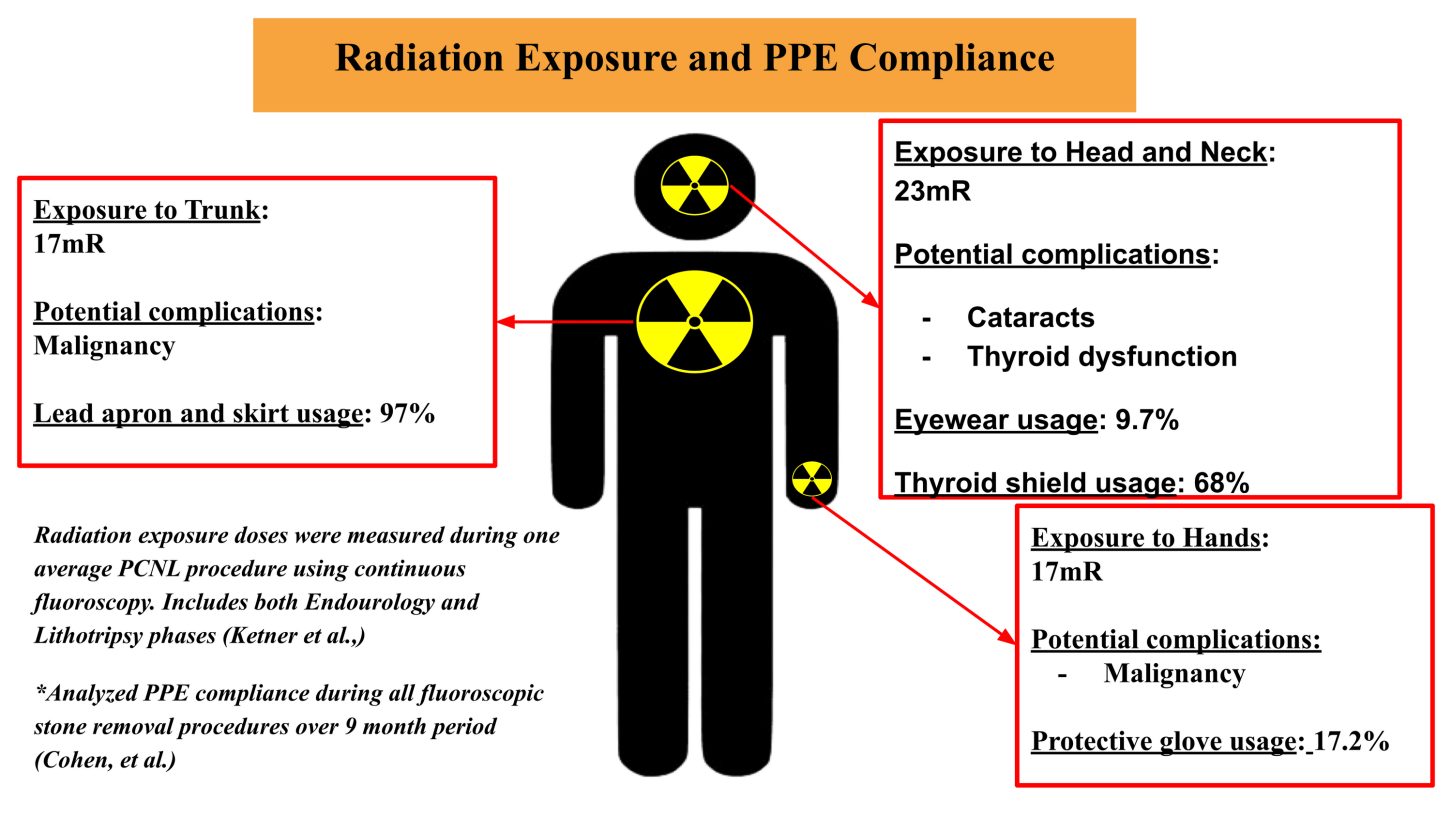
Radiation Exposure and PPE Compliance in the Operating Room Source: [21,28] PPE: personal protective equipment; PCNL: percutaneous nephrolithotomy Image Credits: Shika Veera, William Olive, Kimberly C. Toumazos, Virgil DeMario, Vaishnavi J. Patel, Devki Patel, Young Son, Thomas J. Mueller

Two studies investigated the reduction of radiation exposure related to changes in equipment, specifically the C-arm and the Mini C-arm (Table 3). Two additional studies discussed the benefits of PPE utilization. The studies included radiation exposure data on the eyes, hands, thyroid, and trunk. Data analysis revealed a significant reduction in radiation exposure to all body parts with a change in equipment as well as PPE. In a study involving 49 lithotripsy procedures performed by one surgeon, dosimeter readings were used to assess the benefit of PPE in situations that resulted in increased radiation exposure, such as calculi with large HU values, upper pole location of the stone, or in patients with an elevated BMI [17,27]. One study showed that the utilization of PPE for the eyes, hands, thyroid, and trunk reduced radiation exposure by 90%, 50%, 95%, and 95%, respectively (Figure 3) [8]. Similarly, one study demonstrated that the use of PPE by surgical staff resulted in a significant reduction in radiation exposure of over 70% in all four body regions [27]. In 148 endourological cases, 60 were completed by one surgeon using a C-arm and dosimeters to measure radiation exposure [15]. Alashban and Shubayr [18] reported a substantial reduction in radiation exposure across multiple body regions with the use of PPE, based on dosimetric measurements collected during endourological procedures, providing objective evidence to support the protective benefit of lead-based equipment.

**Table 3:** Radiation Dose Reduction PPE: personal protective equipment

Study	Intervention	Risk			
		Eyes	Hands	Thyroid	Trunk
Alashban & Shubayr [18]	PPE	35-90% reduction of radiation exposure	20-50% reduction of radiation exposure	>95% reduction of radiation exposure	>95% reduction of radiation exposure
Harris et al. [2]	C-arm	90.62% reduction in radiation exposure	90.59% reduction in radiation exposure	-	90.59% reduction in radiation exposure
Kim et al. [21]	PPE	Radiation reduction 70%-92%	Radiation exposure reduced by 76.6%	Radiation exposure reduced by 96%	Radiation exposure reduced by 98%
Sung et al. [14]	Mini C-arm	-	52.74% reduction of radiation exposure	96% reduction of radiation exposure	100% reduction of radiation exposure

Safety measures

Eight studies investigated relevant protocol interventions that resulted in a significant reduction of radiation exposure (Table 4). Data analysis revealed that four of the studies supported the use of pulsed fluoroscopy in endourological procedures. In two studies, the utilization of additional lead shielding was found to have a notable benefit in protection. Overall, changes in protocol that reduced the duration of fluoroscopy were associated with a logically reduced radiation exposure. Blair et al. demonstrated the effectiveness of a reduced fluoroscopy protocol in achieving an 80.8% reduction in fluoroscopy time without compromising surgical outcomes, thereby reinforcing the importance of institutional-level interventions in mitigating radiation risk [25].

**Table 4:** Safety Measures

Study	Safety Measures
Prospective Cohort Studies	
Durutovic et al. [19]	Pulsed fluoroscopy technique resulted in 36.79% reduction in fluoroscopic time
Kendrick et al. [20]	Methods used to reduce radiation exposure: ceiling-mounted hanging lead shield (51%), table-mounted lead (32%) of observed cases, and both shields (24%)
Ozaki et al. [6]	Pulsed fluoroscopy resulted in 50% reduction of radiation exposure
Usawachintachit et al. [5]	Ultrasound-guided protocol had 71.7% improvement in fluoroscopic screening time
Retrospective Cohort Studies	
Blair et al. [25]	Reduced fluoroscopy protocol resulted in 80.8% reduction in fluoroscopy time with similar outcomes
Kiang et al. [11]	Methods used to decrease radiation exposure: reduced fluoroscopy time (88%), reduced exposed area with a diaphragm (75%), radiation source close to the patient (72%), pulsed fluoroscopy (44%)
Survey Studies	
Henderickx et al. [10]	Methods used to decrease radiation exposure: pulsed fluoroscopic procedures (43.5%), used dosimeter (49%), lead screens (1.76%)
Tzelves et al. [13]	Methods used to reduce radiation exposure: use of alarm (39%), use of last image hold (64%), and modification of fluoroscopy machine settings (20%)

Discussion

This systematic review was conducted to assess the amount of radiation exposure to urologists, as well as to evaluate their safety protocols and knowledge regarding exposure. This analysis compared articles according to compliance, knowledge, radiation risk, and safety measures.

It was found that the most worn PPE is a shield that covers the trunk. In terms of education, a 2019 survey sent to all urology program directors and coordinators in the United States (US) reported that only 13% of trainees in the US had received formal radiation safety training [2]. Exclusively, no trainees reported the use of lead-lined gloves or glasses [11,23]. The PPE compliance for the use of thyroid shields was determined to be high, ranging from 68% to 99%. This lack of compliance is surprising given the known association between radiation exposure and thyroid cancer development with a minimum latency period of 5-10 years [15,26,29]. The lack of formal training regarding the risk of radiation exposure could be attributed to the excessive use of fluoroscopy, and as such, older physicians were not given appropriate training on the risks of radiation damage [7,13,30,31]. Another consideration is that the medical staff is not exclusive to physicians; therefore, the other operating room staff may not be experienced or have received adequate training for procedures that involve the use of radiation equipment [9,22].

Currently, the ICRP emphasizes the importance of education and training for all personnel exposed to ionizing radiation, recommending regular education on radiation risks, safety principles, and protective measures, even in the absence of formal mandates [3]. These guidelines underscore the importance of implementing structured radiation safety curricula within urology training programs to foster a culture of safety and compliance. In the future, the ICRP may consider several options to further improve ALARA outcomes. The ICRP could collaborate with leadership organizations that oversee education and training for healthcare workers exposed to radiation to ensure that the risks of radiation are properly taught, thereby increasing compliance and knowledge of PPE. Additional steps could involve ICRP mandating training provided by the hospital system for anyone with potential radiation exposure.

The risk of occupational radiation exposure has increased in urological surgery recently, primarily due to the increased use of fluoroscopy. The risks of radiation exposure include the development of cataracts, radiation-induced skin injury, and malignancy [10,28,32-35]. The utilization of appropriate PPE resulted in an over 95% reduction in intraoperative radiation exposure. PPE, including a lead apron, thyroid shield, lead-lined glasses, lead gloves, and the consistent use of a dosimeter, has been proven effective in reducing radiation exposure, especially among endourologists at high risk of exposure due to upper tract surgeries [21,36,37].

The findings indicate that modifications to existing protocols can be implemented to decrease radiation exposure during upper tract procedures. The most notable advancements include pulsed fluoroscopy, ultrasound guidance, and mini-C-arm. Pulsed fluoroscopy has been shown to reduce radiation exposure by more than 70% (12.5 vs. 3.0 seconds) due to its discontinuous usage, prompting physicians to be more conscientious about using fluoroscopy only when necessary [17,27]. Ultrasound-guided techniques can reduce radiation exposure by using alternative imaging modalities prior to the procedure or can even be used to gain access for PCNL [17,18,38]. Additionally, the mini-C-arm is also favored over the standard C-arm because of the lower amount of radiation exposure produced by the tube generator [14,39]. Compared to the standard fixed C-arm, the mobile C-arm offers more control and flexibility but lacks a powerful X-ray generator, high-quality imaging, and a large field of view. These components are often not necessary during upper tract urological procedures. Additionally, the use of fixed fluoroscopy and standard C-arms has been associated with increased measurable radiation exposure to both patient and surgeon [5,6,19,20,25,40,41].

In addition to protocol-level changes, several practical steps can be taken by community urologists to reduce radiation exposure. These include routine use of personal dosimeters to monitor exposure, reinforcing a culture of safety by incorporating radiation safety checklists into the OR workflow, and encouraging peer-to-peer training or brief in-service sessions on fluoroscopy safety for all staff [8,18]. Community practices can also adopt standardized low-dose fluoroscopy protocols and ensure accessible storage and routine inspection of PPE, which has been shown to improve compliance [8,18]. These strategies are cost-effective, scalable, and can be readily implemented outside of academic centers.

A strength of this study is its comprehensive evaluation of multiple dimensions of radiation safety, allowing for the inclusion of a diverse range of studies that collectively provide a broad and informative perspective on current practices. Conversely, its focus on upper tract urological procedures provides a stronger focus on selective procedures compared to previous studies. The literature search revealed 209 articles that included the specified keywords and MeSH terms; however, 159 of these were excluded from review. If more studies had been previously completed, possibly more high-quality studies could have been found, leading to even clearer conclusions about radiation exposure among urologists.

This review is limited by the relatively low number of studies examining each outcome of interest, as well as heterogeneity in study design, populations, and outcome measures. Additionally, the majority of included studies carried a high risk of bias, and there was a notable lack of randomized controlled trials. While a prospective study could offer higher-quality evidence, the ethical and practical challenges of studying radiation exposure, particularly withholding PPE or safety education, make such trials difficult to implement. These limitations underscore the need for more standardized methodologies and robust study designs in future research on radiation safety in urology.

Longitudinal retrospective studies discussing the outcomes of those who were exposed to radiation in the surgical occupational setting of upper tract urological surgery should be considered as a topic of future study. A quality improvement investigation could be conducted as fluoroscopy protocols undergo modification to reduce radiation risk, assessing cost-effectiveness and patient care outcomes. Although this study focuses on the risk of radiation exposure from the physician and personnel perspectives, it is also important to consider the benefits of reduced radiation to the patient population.

## Conclusions

Based on the findings, there is a significant improvement in radiation exposure reduction with increased training on radiation risk and the use of PPE. There are currently no established guidelines for radiation exposure in upper tract urological surgeries, despite the increasing use of fluoroscopy and the associated risk of radiation exposure. These results indicate that implementing regulatory guidelines for radiation training and exposure reduction should be a priority.

## Appendices

**Table 5:** PRISMA Checklist PRISMA: Preferred Reporting Items for Systematic Reviews and Meta-Analyses

Section and Topic	Item #	Checklist Item
Title		
Title	1	Identify the report as a systematic review.
Abstract		
Abstract	2	See the PRISMA 2020 for Abstracts checklist.
Introduction		
Rationale	3	Describe the rationale for the review in the context of existing knowledge.
Objectives	4	Provide an explicit statement of the objective(s) or question(s) the review addresses.
Methods		
Eligibility criteria	5	Specify the inclusion and exclusion criteria for the review and how studies were grouped for the syntheses.
Information sources	6	Specify all databases, registers, websites, organizations, reference lists and other sources searched or consulted to identify studies. Specify the date when each source was last searched or consulted.
Search strategy	7	Present the full search strategies for all databases, registers and websites, including any filters and limits used.
Selection process	8	Specify the methods used to decide whether a study met the inclusion criteria of the review, including how many reviewers screened each record and each report retrieved, whether they worked independently, and, if applicable, details of automation tools used in the process.
Data collection process	9	Specify the methods used to collect data from reports, including how many reviewers collected data from each report, whether they worked independently, any processes for obtaining or confirming data from study investigators, and, if applicable, details of automation tools used in the process.
Data items	10a	List and define all outcomes for which data were sought. Specify whether all results that were compatible with each outcome domain in each study were sought (e.g. for all measures, time points, analyses), and if not, the methods used to decide which results to collect.
	10b	List and define all other variables for which data were sought (e.g. participant and intervention characteristics, funding sources). Describe any assumptions made about any missing or unclear information.
Study risk of bias assessment	11	Specify the methods used to assess risk of bias in the included studies, including details of the tool(s) used, how many reviewers assessed each study and whether they worked independently, and if applicable, details of automation tools used in the process.
Effect measures	12	Specify for each outcome the effect measure(s) (e.g., risk ratio, mean difference) used in the synthesis or presentation of results.
Synthesis methods	13a	Describe the processes used to decide which studies were eligible for each synthesis (e.g., tabulating the study intervention characteristics and comparing against the planned groups for each synthesis (item #5)).
	13b	Describe any methods required to prepare the data for presentation or synthesis, such as handling of missing summary statistics or data conversions.
	13c	Describe any methods used to tabulate or visually display results of individual studies and syntheses.
	13d	Describe any methods used to synthesize results and provide a rationale for the choice(s). If meta-analysis was performed, describe the model(s), method(s) to identify the presence and extent of statistical heterogeneity, and software package(s) used.
	13e	Describe any methods used to explore possible causes of heterogeneity among study results (e.g. subgroup analysis, meta-regression).
	13f	Describe any sensitivity analyses conducted to assess the robustness of the synthesized results.
Reporting bias assessment	14	Describe any methods used to assess risk of bias due to missing results in a synthesis (arising from reporting biases).
Certainty assessment	15	Describe any methods used to assess certainty (or confidence) in the body of evidence for an outcome.
Results		
Study selection	16a	Describe the results of the search and selection process, from the number of records identified in the search to the number of studies included in the review, ideally using a flow diagram.
	16b	Cite studies that might appear to meet the inclusion criteria but that were excluded, and explain why they were excluded.
Study characteristics	17	Cite each included study and present its characteristics.
Risk of bias in studies	18	Present assessments of risk of bias for each included study.
Results of individual studies	19	For all outcomes, present, for each study: (a) summary statistics for each group (where appropriate) and (b) an effect estimate and its precision (e.g. confidence/credible interval), ideally using structured tables or plots.
Results of syntheses	20a	For each synthesis, briefly summarize the characteristics and risk of bias among contributing studies.
	20b	Present results of all statistical syntheses conducted. If meta-analysis was done, present for each the summary estimate and its precision (e.g. confidence/credible interval) and measures of statistical heterogeneity. If comparing groups, describe the direction of the effect.
	20c	Present results of all investigations of possible causes of heterogeneity among study results.
	20d	Present results of all sensitivity analyses conducted to assess the robustness of the synthesized results.
Reporting biases	21	Present assessments of risk of bias due to missing results (arising from reporting biases) for each synthesis assessed.
Certainty of evidence	22	Present assessments of certainty (or confidence) in the body of evidence for each outcome assessed.
Discussion		
Discussion	23a	Provide a general interpretation of the results in the context of other evidence.
	23b	Discuss any limitations of the evidence included in the review.
	23c	Discuss any limitations of the review processes used.
	23d	Discuss implications of the results for practice, policy, and future research.
Other information		
Registration and protocol	24a	Provide registration information for the review, including the register name and registration number, or state that the review was not registered.
	24b	Indicate where the review protocol can be accessed, or state that a protocol was not prepared.
	24c	Describe and explain any amendments to information provided at registration or in the protocol.
Support	25	Describe sources of financial or non-financial support for the review and the role of the funders or sponsors in the review.
Competing interests	26	Declare any competing interests among the review authors.
Availability of data, code and other materials	27	Report which of the following are publicly available and where they can be found: template data collection forms; data extracted from included studies; data used for all analyses; analytic code; any other materials used in the review.
